# Association of Macrophage Accumulation and Polarization in Patients with Obesity and Diabetes with Diabetic Remission After Bariatric Surgery

**DOI:** 10.1007/s11695-025-08389-0

**Published:** 2025-12-22

**Authors:** Sa-Hong Kim, Ji-Soo Kim, Jaeun Yoo, Kyoungyun Jeong, Jeesun Kim, Yo-Seok Cho, Ji-Hyeon Park, Jaemoon Koh, Seong-Ho Kong, Do-Joong Park, Young-Min Cho, Doo Hyun Chung, Han-Kwang Yang, Hyuk-Joon Lee

**Affiliations:** 1 Department of Surgery, Seoul National University Hospital, Seoul, Republic of Korea; 2 Department of Surgery, Seoul National University College of Medicine, Seoul, Republic of Korea; 3 Kangjin Clinic, Hochimin city, Vietnam; 4 Cancer Research Institute, Seoul National University College of Medicine, Seoul, Republic of Korea; 5 Department of Surgery, Seoul St. Mary’s Hospital, Seoul, Republic of Korea; 6 Department of Pathology, Seoul National University Hospital, Seoul, Republic of Korea; 7 Laboratory of Immune Regulation in Department of Biomedical Sciences, Seoul National University College of Medicine, Seoul, Republic of Korea; 8 Department of Internal Medicine, Seoul National University Hospital, Seoul, Republic of Korea

**Keywords:** Obesity, Diabetes mellitus, type 2, Macrophage, Bariatric surgery

## Abstract

**Introduction:**

Chronically elevated proinflammatory cytokines and following low-grade tissue inflammation in adipose tissue of patients with obesity induced by M1 macrophages may increase insulin resistance and type 2 diabetes. However, actual macrophage distribution in patients with obesity and diabetes has not clearly confirmed yet. We aimed to reveal the relationship of macrophage accumulation and polarization in visceral fat to patients’ obesity and diabetes status, and to validate the role of preoperative M1 macrophage polarization as a predictor for diabetic remission after bariatric surgery.

**Methods:**

This single-center prospective observational study enrolled 80 patients. Experimental and control 1 group included patients with BMI≧30, with/without type 2 diabetes. Control 2 and control 3 group included patients with stage I gastric cancer and BMI<25, with/without type 2 diabetes. Macrophage accumulation and polarization were evaluated from visceral fat, collected during surgery, through histology, flow cytometry, and RT-PCR. Diabetic and inflammatory indices were assessed preoperatively and postoperatively 12 months. Experimental group were additionally analyzed based on postoperative diabetic remission and preoperative ABCD score.

**Results:**

Patients with obesity (experimental and control 1) exhibited higher macrophage accumulation (*p*<0.001), M1 polarization (*p*<0.001), insulin resistance, WBC, CRP, and M1 marker (PELI1) than patients without obesity. Experimental group showed significantly higher macrophage accumulation (*p*<0.001). M1 dominance was observed in patients achieving complete remission postoperatively or with ABCD score≧7 preoperatively.

**Conclusion:**

Patients with obesity and diabetes (experimental) demonstrated macrophage accumulation, M1 polarization, and proinflammatory circumstance. Preoperative M1 dominance with proinflammatory circumstances may be associated with better diabetic remission after bariatric surgery.

**Supplementary Information:**

The online version contains supplementary material available at 10.1007/s11695-025-08389-0.

## Key Points

Patients with obesity had higher macrophage accumulation and M1 polarization in the visceral adipose tissue.

Patients with obesity and diabetes showed M1 polarization and proinflammatory circumstance in the visceral adipose tissue.

Preoperative M1 dominance with proinflammatory circumstance may be related to better diabetic remission after bariatric surgery.

**Supplementary Information:**

The online version contains supplementary material available at 10.1007/s11695-025-08389-0.

## Introduction

Type 2 Diabetes Mellitus (T2DM) is a multifactorial metabolic disorder characterized by insulin resistance and impaired beta-cell function resulting in impaired glucose homeostasis. Obesity is closely associated with insulin resistance manifesting decreased insulin-regulated glucose transport and metabolism in adipocytes and skeletal muscle, along with impaired suppression of hepatic glucose output [1].

Adipose tissue is no longer regarded as merely a passive energy reservoir but rather as an active endocrine and immunometabolic organ that secretes a wide spectrum of bioactive molecules known as adipokines, encompassing both pro- and anti-inflammatory cytokines such as leptin, serum amyloid A (SAA), tumor necrosis factor-alpha (TNF-α), plasminogen activator inhibitor-1 (PAI-1), adipsin, resistin, chemerin, and adiponectin [2–13]. These adipokines play pivotal roles in regulating systemic inflammation, vascular homeostasis, and insulin sensitivity. In particular, leptin promotes macrophage activation, endothelial dysfunction, and insulin resistance, whereas adiponectin exerts anti-inflammatory and insulin-sensitizing effects [2]. Serum amyloid A (SAA), an acute-phase reactant produced by both hepatocytes and adipocytes, is markedly elevated in morbid obesity and decreases after bariatric surgery, underscoring its role as a metabolic link between inflammation and insulin resistance [3]. In addition, adipose tissue serves as a source of vasoactive and immunomodulatory factors, including leptin and SAA, that influence local microcirculation and immune cell recruitment [4, 5]. Other adipokines, including resistin [6], chemerin [7, 8], and asprosin [9, 10], also contribute to immune–metabolic crosstalk and glucose dysregulation. As adiposity increases, these alterations of adipokines collectively shift toward a pro-inflammatory profile, establishing a state of chronic low-grade inflammation that drives insulin resistance and the pathogenesis of obesity-related disorders such as hypertension, dyslipidemia, and type 2 diabetes [11, 12].

Notably, adipose tissue macrophages (ATMs), the predominant immune cells residing in adipose tissue, have been emerged as critical regulators for those cytokine-mediated metabolic processes [14, 15]. ATMs can locally proliferate and accumulate forming crown-like structures (CLS), which are clusters of ATMs surrounding dead adipocytes [16]. Various microenvironmental stimuli can induce the ATMs to express distinct phenotypes and physiological activities, known as macrophage polarization [17–20]. In patients with obesity, ATMs tend to polarize into classically activated (M1) macrophages which is characterized by proinflammatory features by producing proinflammatory cytokines such as TNF-α, interleukin-1 (IL-1), interleukin-6 (IL-6) and nitric oxide [21] via inducible nitric oxide synthase (iNOS), while in lean individuals, ATMs tend to polarize into alternatively activated (M2) macrophages which express anti-inflammatory features through interleukin-10 (IL-10) and arginase-1 (ARG1) [17, 21, 22]. Chronically elevated proinflammatory cytokines and following low-grade inflammation in adipose tissue of patients with obesity induced by M1 macrophages have been demonstrated correlative and causative relationship to insulin resistance and type 2 diabetes mellitus development [15, 23–29]. However, conversely, the actual distribution of macrophages particularly in patients with obesity and diabetes remains unclear.

Meantime, with increasing prevalence of patients with severe obesity suffering from intractable type 2 diabetes, bariatric surgery has gained attention for its effectiveness in achieving diabetic remission and reducing obesity-related comorbidities [30]. While clinical parameters, such as ABCD (Age, BMI, C-peptide, and Duration of diabetes) score, IMS (Individualized Metabolic Surgery) score, DiaRem (Diabetes Remission) score, and Ad-DiaRem (Advanced DiaRem) score, have been developed to predict the effectiveness of diabetic treatment, their predictive power for surgically-treated patients remains still on debate [31–34].

Based on this background, we hypothesize that obesity and diabetes can induce distributional change in macrophages and inflammatory circumstance in human visceral fat, which may be associated with metabolic outcomes after bariatric surgery.

Accordingly, this study investigated macrophage accumulation and polarization in visceral fat in relation to patient’s presence of obesity and/or diabetes, and explored their potential role as a preoperative predictor for diabetic remission after bariatric surgery.

## Materials and Methods

### Patients

This single-center prospective observational study enrolled patients in our hospital from November 2019 to August 2020. Patients with obesity (body mass index; BMI≧30 kg/m²) undergoing bariatric surgery and patients without obesity (BMI<25 kg/m²) undergoing gastric cancer surgery for stage I early gastric cancer (EGC), confined to mucosa or submucosa, were enrolled. To minimize any potential bias derived from inflammatory circumstance, patients with deeper invasion beyond the muscularis propria, lymph node metastasis, experience of prior inflammation-inducing procedures such as endoscopic submucosal dissection (ESD), or neoadjuvant chemotherapy were excluded. Given the paucity of clinical evidence that cancer induces obesity or metabolic alterations, and the fact that EGC follows a slow and biologically indolent course, remaining asymptomatic for prolonged periods without notable weight loss or energy wasting attributable to disease activity [35–38], patients with stage I EGC without obesity were therefore considered an appropriate and metabolically stable control group for investigating adipose tissue biology.

The patients were categorized into four groups: experimental group (patients with obesity and diabetes), control 1 group (patients with obesity but without diabetes), control 2 group (patients without obesity but with diabetes, stage I EGC), and control 3 group (patients with neither obesity nor diabetes, stage I EGC). Only patients with type 2 diabetes were included.

Baseline characteristics, including age, sex, body weight, height, body mass index (BMI), diabetic status, diabetic duration (year), the number of diabetic medications were collected.

The study followed the Declaration of Helsinki and was approved by the Institutional Review Board (IRB) (No.: H-1909-061-1064) of our hospital. The written informed consent was obtained from all participants.

### Adipose Tissue Collection and Stromal Vascular Fraction (SVF) Cell Preparation

Over 60 g of visceral adipose tissue was obtained from the patient’s greater omentum during surgery. The fat tissue was chopped into 1 mm x 1 mm pieces, divided into six tubes (10 g each), and treated with 0.1% collagenase buffer (RPMI with collagenase Type 1, Worthington Biochemical Corporation, USA; Cat. No. LS004194) in a shaking incubator (150 RPM, 60 min, 37 °C). The collagen-digested mixture was filtered and centrifuged (2000 RPM, 5 min, 4˚C) to isolate stromal vascular fraction (SVF) cells. SVF cells were treated with RBC lysis solution and stored at −80 °C.

### Flow Cytometry

Alexa Fluor 700, a near-infrared dye, was conjugated with anti-human CD45 (immune cell marker; BD Biosciences, USA; RRID: AB_1645452), FITC (fluorescein isothiocyanate) with anti-human CD14 (macrophage marker; BD Biosciences, USA; RRID: AB_395798), PE-Cy7 (phycoerythrin-Cyanine7) with anti-human CD11c (M1 marker; BD Biosciences, USA; RRID: AB_10611859), and PE (phycoerythrin) with anti-human CD163 (M2 marker; BD Biosciences, USA; RRID: AB_396296). These dye-conjugated markers were incubated with SVF cells in a light-blocked box at 4 °C for 30 min [39]. After washing, centrifuging, and fixation, SVF cells were analyzed with a BD FACSCanto™ Flow Cytometer (BD Biosciences, USA; RRID: SCR_018055) and BD FACSDiva™ software (BD Biosciences, USA; RRID: SCR_001456). The proportion of macrophage, M1, and M2 in SVF (% of SVF) was measured, and M1/M2 ratio was separately calculated.

### Real-Time Quantitative Reverse Transcription Polymerase Chain Reaction (Real-Time qRT-PCR)

Complementary DNA was synthesized from SVF cell RNA with TOPscript™ cDNA Synthesis kit (Enzynomics, South Korea; Cat. No. EZ0005M), and real-time qRT-PCR was conducted with StepOnePlus™ Real-Time PCR System (Applied Biosystems™, USA; RRID: SCR_015805).

Relative expression levels of macrophage indicator genes, including Pellino E3 Ubiquitin Protein Ligase 1 (*PELI1*), whose protein product functions as a cytosolic regulator of M1 polarization contributing to glucose intolerance in obesity [40], and *NOS2* [41] (a proinflammatory M1 indicator gene), and *ARG1* (an anti-inflammatory M2 indicator gene) [22], were compared to glyceraldehyde-3-phosphate dehydrogenase (*GAPDH*) gene, a housekeeping gene used as the reference. Gene expression levels of macrophage indicator genes are presented as 2-ΔCt values.

### Microscopic Evaluation

Hematoxylin and eosin (H&E)-stained paraffin blocks were reviewed under low-power field (LPF) to determine crown-like structure (CLS) count, and anti-human CD68 antibody-stained blocks were reviewed with immunohistochemistry (IHC) under high-power field (HPF) for macrophage count. Counts were defined as the average from at least two random points, and evaluations were conducted by pathologists in a blinded fashion.

### Laboratory Tests

The study measured diabetes-related parameters including fasting glucose, HbA1c, fasting insulin, C-peptide, and Homeostatic Model Assessment for Insulin Resistance (HOMA-IR) [42], and inflammatory parameters including white blood cell (WBC) count, the proportion (%) of neutrophil, lymphocyte, and monocyte, neutrophil-to-lymphocyte ratio, and C-reactive protein (CRP). All laboratory tests were conducted both preoperatively and at one year postoperatively, using blood samples collected under standardized fasting conditions (minimum 8 h) withholding all medications prior to sampling.

### Evaluation of Postoperative Diabetic Remission

Within experimental group, complete remission was defined as HbA1c < 6.0% (42mmol/mol) and fasting glucose level < 100 mg/dL (5.6mmol/L) without anti-diabetic medication at the point of postoperative one year. Partial remission was defined as HbA1c < 6.5.% (48mmol/mol) and/or fasting glucose level between 100 and 125 mg/dL (5.6–6.6.9mmol/L) without anti-diabetic medication at the point of postoperative one year. Improved disease encompassed the patients with reduced HbA1c and fasting glucose not fulfilling diabetic remission criteria. ‘All remission group’ comprised complete remission and partial remission group [43].

### ABCD Score Threshold for Experimental Group

The ABCD score is composed of the patient’s age, body mass index (BMI), C-peptide level, and duration of diabetes (year) [31]. Patients in ‘all remission group’ were subdivided into two groups based on threshold of ABCD score 7; ABCD ≧ 7 group and ABCD < 7 group. Preoperative baseline characteristics and macrophage-related parameters of both groups were evaluated.

### Statistics

The distribution of each baseline characteristic was first assessed using the Shapiro–Wilk test, a statistical test of normality. Variables fulfilling the assumption of normality (parametric) are reported as mean ± standard deviation, whereas those not meeting this assumption (non-parametric) are reported as median with interquartile range (IQR).

Group comparisons among four groups were conducted using two-way ANOVA for parametric variables and the Scheirer–Ray–Hare test, a widely used non-parametric analogue to two-way ANOVA, for non-parametric variables. For each variable, *p*-values were obtained for the main effect of obesity, the main effect of diabetes, and the interaction effect between obesity and diabetes.

Additional subgroup comparisons, namely between complete remission and partial remission and between ABCD≧7 group and ABCD<7 group, were conducted using appropriate two-sample tests according to distributional characteristics. A *p*-value < 0.05 was considered statistically significant.

SPSS software 21.0 (IBM corporation, USA; RRID: SCR_002865), SAS 9.4 (SAS institute, USA; RRID: SCR_008567), and Prism 8.0 (GraphPad, USA; RRID: SCR_002798) were used for analyses and graphical illustrations.

## Results

### Baseline Characteristics

A total of 80 patients were enrolled and divided into four groups of 20 patients each.

Patients with diabetes (experimental group and control 2 group) showed higher median fasting glucose and median HbA1c level than those without diabetes (control 1 group and control 3 group) (all *p*<0.001).

Patients with obesity (experimental group and control 1 group) demonstrated higher fasting insulin, C-peptide, and HOMA-IR than patients without obesity (control 2 group and control 3 group) (all *p*<0.001).

Among the inflammatory markers, patients with obesity exhibited higher WBC and CRP than patients without obesity (all *p*<0.001) (Table 1).

**Table 1:** Baseline characteristics

Characteristics		Experimental (*N* = 20)	Control 1 (*N* = 20)	Control 2 (*N* = 20)	Control 3 (*N* = 20)	*p* value ^a^	*p* value^b^	*p* value^c^
Age (year) *		39.45 ± 11.99	32.70 ± 10.82	69.55 ± 5.91	57.95 ± 9.58	< 0.001	< 0.001	0.274
Sex	Male (%)	10 (50.0)	6 (30.0)	15 (75.0)	8 (40.0)			
	Female (%)	10 (50.0)	14 (70.0)	5 (25.0)	12 (60.0)			
BMI (kg/m^2^) ^†^		39.55 (36.94–44.17)	38.90 (36.61–41.70)	22.27 (21.44–23.92)	23.65 (22.83–24.13)	< 0.001	0.553	0.193
Surgery type	Sleeve gastrectomy (%)	17 (85.0)	18 (90.0)	0	0			
	Gastric bypass (%)	3 (15.0)	2 (10.0)	0	0			
	Total gastrectomy (%)	0	0	0	5 (25.0)			
	Distal gastrectomy (%)	0	0	15 (75.0)	10 (50.0)			
	Pylorus-preserving gastrectomy (%)	0	0	3 (15.0)	5 (25.0)			
	Proximal gastrectomy (%)	0	0	2 (10.0)	0			
**Diabetic Features**								
Fasting glucose (mg/dL) ^†^		137.50 (116.00–200.25)	98.00 (93.00–109.50)	127.00 (113.00–143.50)	95.00 (86.00–103.75)	0.486	< 0.001	0.213
HbA1c (%) ^†^		7.70 (6.57–9.10)	5.65 (5.10–5.80)	7.00 (6.65–7.25)	5.60 (5.45–5.70)	0.574	< 0.001	0.467
Fasting insulin (µIU/ml) ^†^		17.00 (11.70–24.25)	20.00 (16.55–26.20)	7.15 (5.90–9.17)	7.90 (6.05–10.50)	< 0.001	0.104	0.480
C-peptide (ng/ml) ^†^		3.65 (2.26–5.12)	3.25 (2.55–4.05)	1.65 (0.95–2.22)	1.20 (0.95–1.35)	< 0.001	0.180	0.637
HOMA-IR ^†^		5.58 (3.44–8.73)	4.90 (3.95–5.88)	2.56 (1.62–3.06)	1.76 (1.52–2.63)	< 0.001	0.511	0.719
Diabetic duration (year)		2.50 (0.50–4.00)		10.00 (4.50–16.25)		0.001	-	-
The number of diabetic medication		2.00 (0.00–2.00)		2.00 (1.00–2.00)		0.068	-	-
Insulin use	yes	5		2				
	no	15		18				
**Inflammatory markers**								
WBC (10^3^/µL) *		9.26 ± 1.84	8.50 ± 1.52	6.47 ± 1.58	5.86 ± 1.36	< 0.001	0.057	0.842
Neutrophil (%) *		61.68 ± 6.82	59.99 ± 5.76	61.31 ± 6.06	57.86 ± 11.20	0.474	0.163	0.627
Lymphocyte (%) *		29.46 ± 6.58	30.27 ± 5.50	28.02 ± 5.35	32.96 ± 10.27	0.660	0.097	0.221
Neutrophil/lymphocyte ratio ^†^		2.01 (1.66–2.55)	2.11 (1.54–2.37)	2.21 (1.79–2.74)	1.71 (1.29–2.89)	0.739	0.130	0.343
Monocyte (%) *		5.90 ± 1.37	6.57 ± 1.64	7.83 ± 2.40	6.43 ± 1.53	0.041	0.405	0.012
C-reactive protein (mg/L) ^†^		0.46 (0.26–1.14)	0.64 (0.37–0.78)	0.04 (0.03–0.10)	0.04 (0.03–0.07)	< 0.001	0.856	0.643
**Macrophage accumulation**								
CLS count (count/low power field) ^†^		2.00 (1.25–3.00)	0.33 (0.00–0.91)	0.00 (0.00–0.00)	0.00 (0.00–0.00)	< 0.001	0.015	0.011
Macrophage count (count/high power field) ^†^		14.67 (10.92–18.75)	8.17 (5.50–10.41)	7.58 (5.46–9.00)	4.42 (1.00–6.33)	0.032	0.148	0.276
**Macrophage polarization**								
Macrophage (% of total SVF) *		5.97 ± 2.42	6.94 ± 2.92	3.30 ± 1.23	3.72 ± 1.60	< 0.001	0.156	0.558
M1 (% of total SVF) ^†^		1.18 (0.91–2.18)	1.69 (1.13–2.24)	0.92 (0.63–1.34)	0.90 (0.74–1.55)	< 0.001	0.673	0.664
M2 (% of total SVF) ^†^		1.48 (0.89–2.16)	2.20 (1.62–3.84)	0.96 (0.51–1.70)	1.12 (0.62–1.87)	0.002	0.082	0.004
M1/M2 ^†^		1.05 (0.52–2.40)	0.67 (0.34–1.08)	0.79 (0.43–2.16)	0.71 (0.46–1.74)	0.862	0.403	0.244
**Macrophage indicator genes**								
*PELI1* ^†^		0.0666 (0.0504–0.0967)	0.0620 (0.0331–0.1069)	0.0298 (0.0203–0.0498)	0.0419 (0.0340–0.0556)	0.002	0.649	0.005
*NOS2* ^†^		0.0005 (0.0003–0.0006)	0.0002 (0.0002–0.0004)	0.0003 (0.0001–0.0004)	0.0002 (0.0001–0.0004)	< 0.001	0.043	0.003
*ARG1* ^†^		0.0003 (0.0002–0.0005)	0.0004 (0.0003–0.0009)	0.0002 (0.0002–0.0005)	0.0001 (0.0001–0.0004)	0.002	0.198	0.041

*BMI* body mass index, *HOMA-IR* Homeostatic Model Assessment for Insulin Resistance, *CLS* crown-like structure

* Variables that fulfilled the assumption of normality (parametric), presented as mean ± standard deviation

† Variables that did not fulfill the assumption of normality (non-parametric), presented as median (interquartile range)

*p*-values were derived from 2-way ANOVA (parametric) or Scheirer–Ray–Hare test (non-parametric)

^a^ Comparison between patients with obesity (experimental and control 1) and patients without obesity (control 2 and control 3) to show the effect of obesity

^b^ Comparison between patients with diabetes (experimental and control 2) and patients without diabetes (control 1 and control 3) to show the effect of diabetes

^c^ Comparison among the groups to show the interaction effect between obesity and diabetes

### Macrophage Accumulation in the Adipose Tissue

**Fig. 1:**
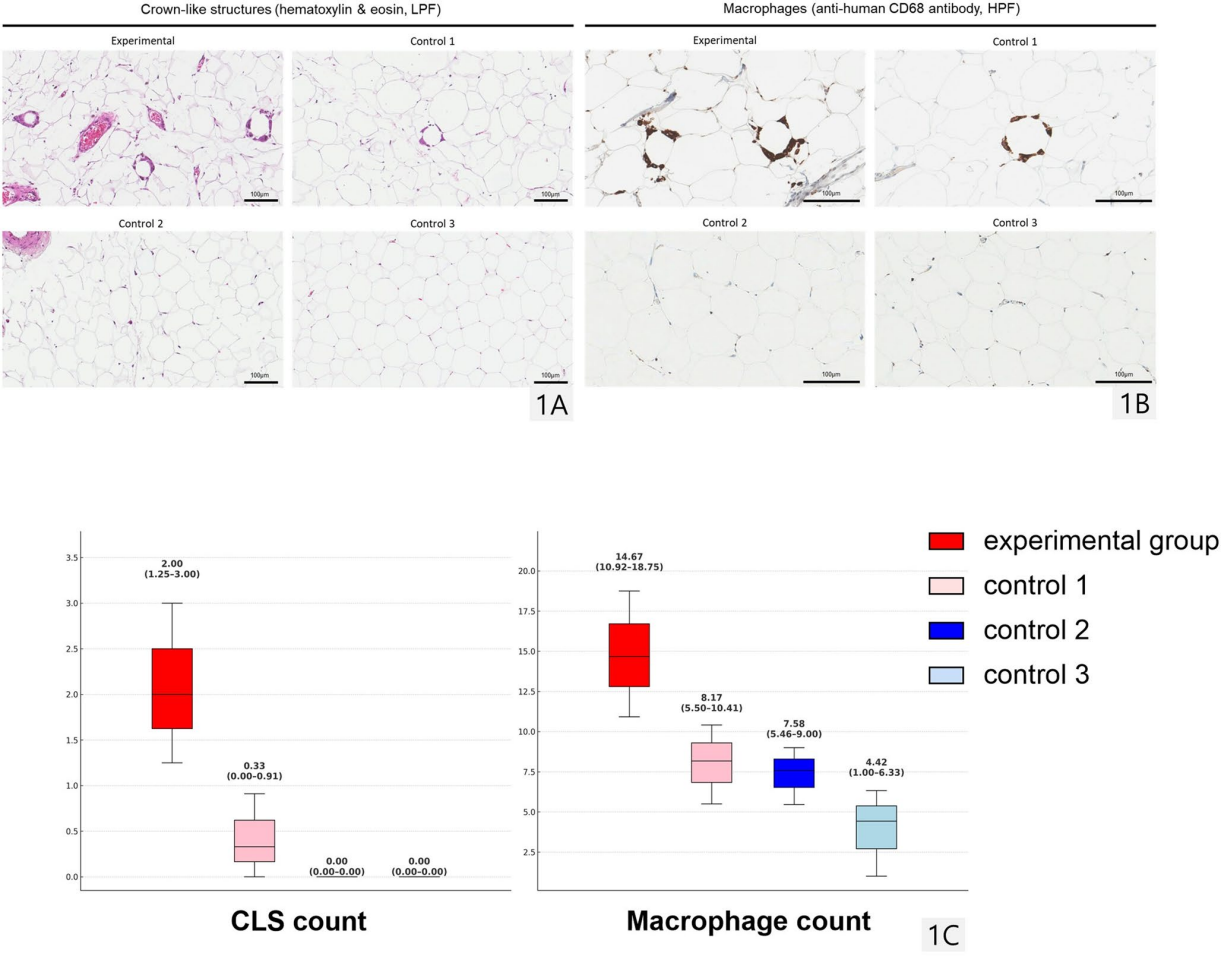
Macrophage accumulation in the adipose tissue The number of crown-like structures (1**A**; hematoxylin & eosin stain; magnification, ×100; scale bar, 100 μm) and macrophages (1**B**; immunohistochemistry stain, anti-human CD68 antibody; magnification, ×400; scale bar, 100 μm) were microscopically reviewed. The numbers above the box plots represent the median with interquartile range (IQR) for each group. The experimental group showed the highest median crown-like structure (CLS) count and macrophage count. *LPF*, low power field; *HPF*, high power field

The experimental group showed the highest median crown-like structure (CLS) count (effect of obesity, *p*<0.001; interaction effect between obesity and diabetes, *p*=0.011) and macrophage count (effect of obesity, *p*=0.032) (Table 1; Fig. 1A-C).

### Macrophage Polarization in the Adipose Tissue

**Fig. 2:**
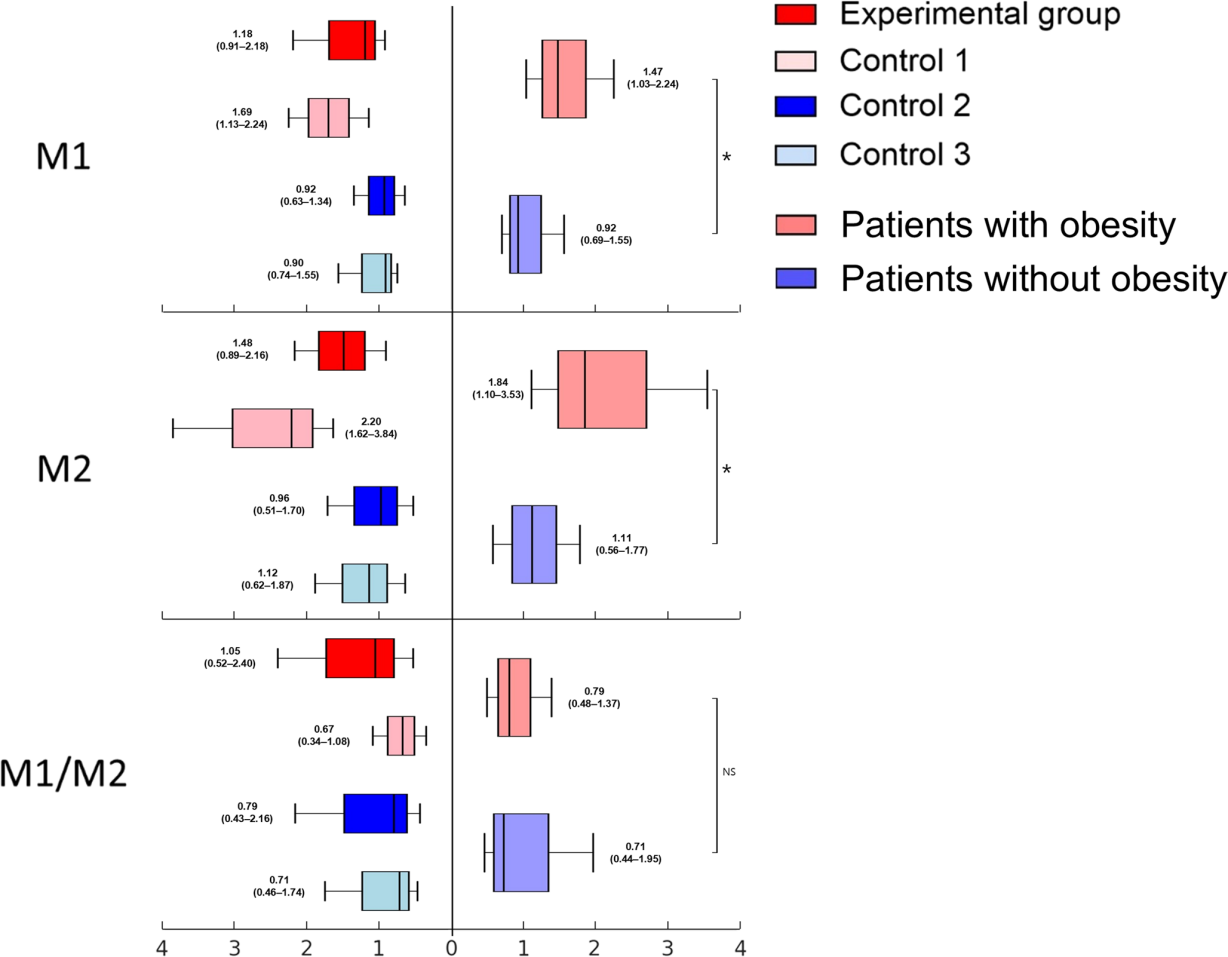
Macrophage polarization in the adipose tissue The proportion of M1, and M2 in stromal vascular fraction cells (% of SVF) was evaluated with flow cytometry, and M1/M2 ratio was separately calculated. The numbers beside the box plots represent the median with interquartile range (IQR) for each group. Patients with obesity (experimental and control 1) exhibited higher M1 polarization. *; statistically significant, NS; not statistically significant

The median proportion of macrophages, M1, and M2 in SVF (% of SVF) were significantly higher in patients with obesity than in patients without obesity (effect of obesity: *p*<0.001, <0.001, and 0.002, respectively; Table 1; Fig. 2).

M1/M2 ratio was the highest in experimental group, but the difference was not statistically significant compared to the other groups (effect of obesity, *p* = 0.862; effect of diabetes, *p* = 0.403; interaction effect between obesity and diabetes, *p* = 0.244) (Table 1; Fig. 2).

### Macrophage Indicator Genes in the Adipose Tissue

The median level of *PELI1* and *NOS2* expression was higher in patients with obesity than in patients without obesity, especially in experimental group (effect of obesity, *p*=0.002, <0.001; interaction effect between obesity and diabetes, *p*=0.005, 0.003).

**Fig. 3:**
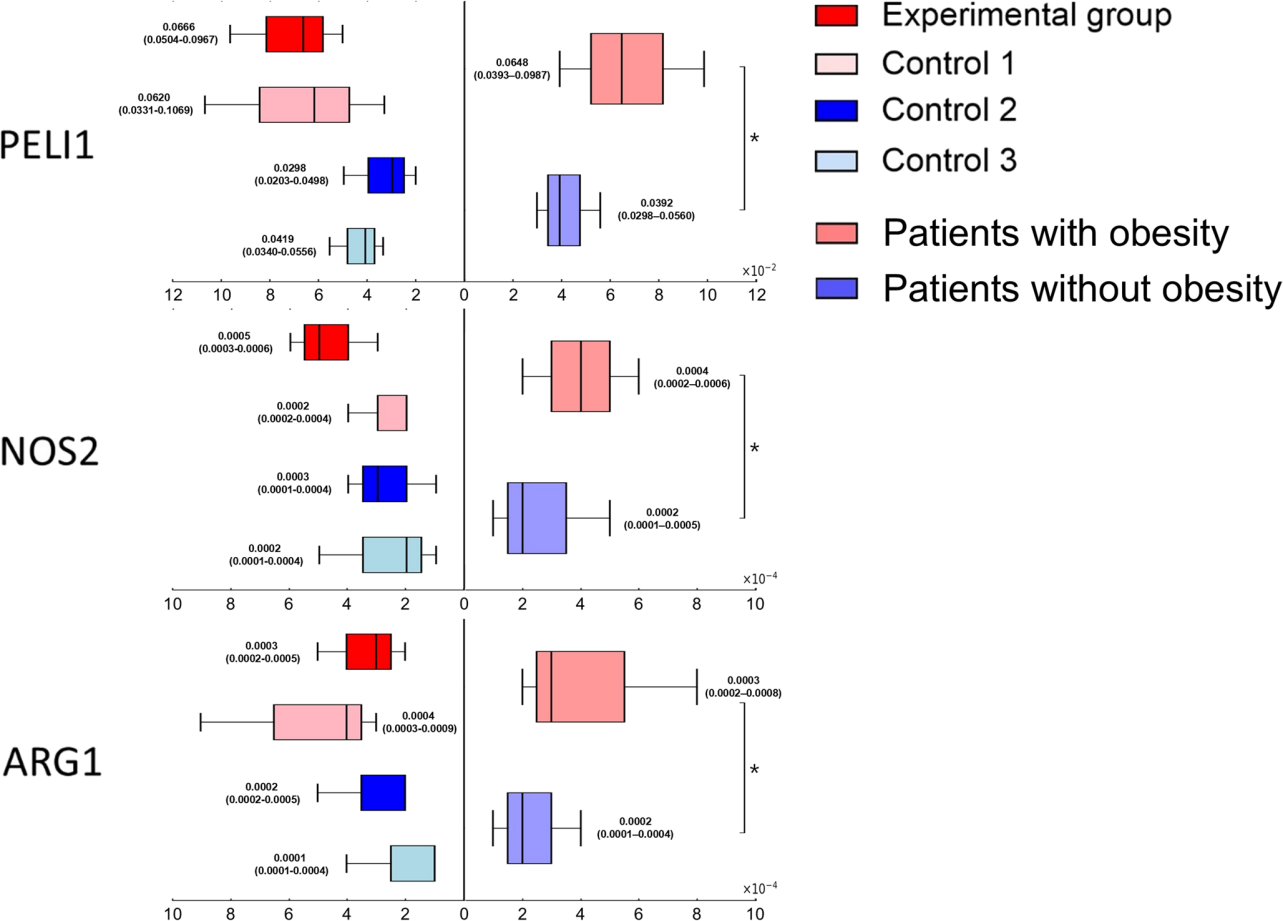
Macrophage indicator genes in the adipose tissue The relative expressions of macrophage indicator genes were evaluated with real-time quantitative reverse transcription polymerase chain reaction (real-time qRT-PCR). The numbers beside the box plots represent the median with interquartile range (IQR) for each group). *; statistically significant,* NS; not statistically significant*

The median level of *ARG1* expression was higher in patients with obesity than in patients without obesity, especially in control 1 group (effect of obesity, *p*=0.002; interaction effect between obesity and diabetes, *p* = 0.041) (Table 1; Fig. 3).

### The Characteristics of the Diabetic Remission Patients of Experimental Group

Experimental group was divided into complete remission group (*N* = 5), partial remission group (*N* = 4), and improved disease group (*N* = 7), with a 20% follow-up loss (4 out of 20 patients) at postoperative one year.

Complete remission group (*N* = 5) showed higher HbA1c and WBC count than partial remission group (*p*=0.048 and 0.025), but fasting glucose and parameters of insulin resistance (fasting insulin, C-peptide, and HOMA-IR) showed no significant differences. Complete remission group consistently showed higher values than the partial remission group in macrophage accumulation, M1 polarization, and M1 indicator genes, except for NOS2, but the differences were not statistically significant.

All remission group (*N* = 9) was subdivided based on their preoperative ABCD score. ABCD≧7 group (*N* = 5) had higher fasting insulin and HOMA-IR than ABCD<7 group (*N* = 4) (*p*=0.029 and 0.057). Among the inflammatory markers, ABCD≧7 group exhibited higher proportion of neutrophil, and neutrophile/lymphocyte ratio, and lower proportion of lymphocyte than ABCD<7 group (*p*=0.087, 0.036, and 0.036). ABCD≧7 group showed similar trends to complete remission group in terms of macrophage accumulation, M1 polarization, and M1 indicator genes, but the differences were not statistically significant (Table 2).

**Table 2:** Baseline characteristics of experimental group according to remission status and ABCD score

Characteristics		Complete Remission (*N* = 5)	Partial Remission (*N* = 4)	*p* value ^a^	All Remission (*N* = 9)		*p* value ^b^
					ABCD ≧ 7 (*N* = 5)	ABCD < 7 (*N* = 4)	
Age (year)		42.00 (33.00–46.00)	48.50 (41.25–55.00)	0.556	36.00 (33.00–42.00)	52.00 (45.25–61.25)	0.063
Sex	Male (%)	3 (60.0)	3 (75.0)				
	Female (%)	2 (40.0)	1 (25.0)				
BMI (kg/m^2^)		36.97 (36.84–40.15)	38.86 (38.63–39.69)	0.556	40.15 (38.94–41.92)	37.57 (36.75–38.33)	0.111
Surgery type	Sleeve gastrectomy (%)	5 (100.0)	4 (100.0)				
	Gastric bypass (%)	0 (0)	0 (0)				
**Diabetic features**							
Fasting glucose (mg/dL)		117.00 (106.00–119.00)	119.00 (98.75–127.00)	1.000	119.00 (113.00–133.00)	111.50 (93.50–119.00)	0.413
HbA1c (%)		7.30 (6.80–7.40)	6.40 (6.30–6.53)	0.048	6.50 (6.50–7.40)	6.70 (6.52–6.92)	1.000
Fasting insulin (µIU/ml)		23.50 (18.70–24.50)	15.90 (15.25–19.95)	0.571	24.25 (23.88–25.55)	15.25 (13.30–16.60)	0.029
C-peptide (ng/ml)		4.90 (3.50–5.00)	3.65 (2.10–7.12)	1.000	5.10 (5.00–5.20)	2.60 (2.10–3.55)	0.032
HOMA-IR		5.40 (4.54–6.90)	2.52 (2.36–5.20)	0.393	7.39 (6.31–8.16)	2.62 (2.44–3.39)	0.057
Diabetic duration (year)		2.50 (2.00–3.00)	3.00 (2.31–4.25)	0.371	3.00 (2.00–3.00)	2.75 (1.94–3.00)	0.905
The number of diabetic medication		1.00 (0.00–2.00)	1.50 (0.75–2.25)	0.610	1.00 (0.00–2.00)	1.50 (0.75–2.25)	0.730
Insulin use	yes	0	0				
	no	5	4				
**Inflammatory markers**							
WBC (10^3^/µL)		10.66 (9.90–11.11)	8.50 (7.58–8.77)	0.025	10.66 (8.79–11.11)	8.64 (7.80–9.04)	0.190
Neutrophil (%)		66.70 (60.20–70.48)	56.25 (55.55–59.08)	0.486	66.50 (63.00–70.40)	55.90 (53.85–56.25)	0.087
Lymphocyte (%)		24.65 (19.52–30.52)	31.90 (29.92–33.17)	0.343	25.50 (20.30–29.00)	35.10 (33.75–35.30)	0.036
Neutrophil/lymphocyte ratio		2.83 (2.00–3.64)	1.73 (1.69–1.95)	0.486	2.61 (2.17–3.48)	1.59 (1.54–1.66)	0.036
Monocyte (%)		6.00 (5.95–6.25)	7.10 (5.38–8.55)	1.000	6.00 (5.80–7.00)	6.00 (5.20–7.35)	1.000
C-reactive protein (mg/L)		0.11 (0.11–0.11)	0.32 (0.24–0.82)	0.533	0.10 (0.07–0.21)	0.33 (0.23–1.31)	0.200
**Macrophage accumulation**							
CLS count (count/low power field)		3.00 (1.50–3.50)	1.83 (0.58–3.25)	0.459	3.00 (1.50–3.00)	2.75 (1.21–4.25)	0.905
Macrophage count (count/high power field)		18.50 (18.00–18.50)	12.00 (8.62–16.25)	0.389	18.50 (14.67–19.00)	13.67 (8.62–18.12)	0.286
**Macrophage polarization**							
Macrophage (% of total SVF)		7.80 (4.20–8.10)	4.60 (4.12–5.47)	0.461	7.80 (4.70–8.10)	4.35 (4.08–5.33)	0.413
M1 (% of total SVF)		1.34 (1.13–4.02)	0.89 (0.86–0.92)	0.063	1.34 (0.99–4.02)	0.90 (0.87–0.96)	0.190
M2 (% of total SVF)		0.94 (0.50–1.82)	1.48 (1.24–2.59)	0.413	0.97 (0.50–1.33)	1.72 (1.45–2.74)	0.286
M1/M2		1.99 (1.21–3.64)	0.61 (0.46–0.75)	0.111	1.99 (1.02–3.64)	0.52 (0.40–0.72)	0.063
**Macrophage indicator genes**							
*PELI1*		0.1010 (0.0851–0.1224)	0.0483 (0.0334–0.0831)	0.200	0.1118 (0.0695–0.1469)	0.0619 (0.0483–0.0761)	0.393
*NOS2*		0.0003 (0.0002–0.0005)	0.0007 (0.0006–0.0008)	0.114	0.0006 (0.0003–0.0007)	0.0005 (0.0005–0.0006)	0.786
*ARG1*		0.0002 (0.0001–0.0006)	0.0007 (0.0005–0.0026)	0.343	0.0005 (0.0003–0.0008)	0.0003 (0.0002–0.0041)	1.000

Results are reported as median (interquartile range)

*BMI*, body mass index; *HOMA-IR*, Homeostatic Model Assessment for Insulin Resistance; *CLS*, crown-like structure.

^a^ *p*-value between complete remission and partial remission group

^b^ *p*-value between ABCD ≧ 7 and ABCD < 7 group

## Discussion

It could be anticipated that the patients with diabetes (experimental group and control 2 group) exhibited elevated levels of fasting glucose and HbA1c. We expected that parameters of insulin resistance, including the levels of fasting insulin, C-peptide, and HOMA-IR, would be also elevated in these patients with diabetes correspondingly, but the trends were different from the expectations. The levels of fasting insulin, C-peptide, and HOMA-IR were significantly higher in patients with obesity (experimental group and control 1 group). Especially looking into control 1 group (patients with obesity but without diabetes) and control 2 group (patients without obesity but with diabetes), control 1 group exhibited elevated insulin resistance parameters despite absence of diabetes, while control 2 group demonstrated low insulin resistance parameters despite having diabetes. It can be inferred that obesity can have considerable effect on elevating insulin resistance.

Our study first confirmed a significantly high level of macrophage accumulation in the visceral adipose tissue of the patients with obesity and diabetes (experimental group), which was obtained during the surgery. Flow cytometric analysis revealed a notably elevated M1/M2 ratio in the experimental group, indicating M1 macrophage polarization. Additionally, patients with obesity (experimental and control 1 groups) showed higher levels of *PELI1*, *NOS2*, WBC, and CRP compared to patients without obesity.

Considering aforementioned elevated insulin resistance parameters of the patients with obesity (fasting insulin, C-peptide, and HOMA-IR), along with M1 polarization with elevated level of WBC, and CRP, we could assume that M1 polarization with the proinflammatory circumstance in adipose tissue of patients with obesity can have correlative and causative role in escalating insulin resistance, consequently increasing the likelihood of future diabetic development [44–46].

Both complete remission and partial remission groups were subsets of the experimental group, which overall exhibited M1 dominance. Interestingly, the complete remission group specifically showed numerical M1 dominance, whereas the partial remission group showed numerical M2 dominance. Additionally, the complete remission group had significantly higher preoperative HbA1c and WBC counts compared to the partial remission group. We further subdivided all remission group (*N* = 9) into two subgroups based on preoperative ABCD score (ABCD≧7 and ABCD<7). The ABCD≧7 group, presumed to have better prognosis, exhibited numerical M1 dominance with higher levels of fasting insulin, HOMA-IR, neutrophil count, and neutrophil-to-lymphocyte ratio, along with lower lymphocyte counts while ABCD<7 group showed numerical M2 dominance. These exploratory findings suggested that heterogeneity may exist even within the experimental group, despite its overall M1 dominance with proinflammatory features. Although limited by small sample size, patients whose preoperative characteristics tended to align more closely with M1 dominance with proinflammatory features appeared more likely to exhibit favorable postoperative outcomes, warranting further investigation in larger cohorts to establish a basis for predictive modeling.

There are some limitations for this study.

First, the one-year follow-up period was insufficient to apply the general definition of diabetic remission, as our analyses were based on diabetic medication cessation at the point of postoperative one-year. Future research with a longer surveillance may be necessary to strengthen the reliability of the study.

Second, the variability of surgical techniques, including both gastric cancer surgeries and bariatric surgeries, could compromise the homogeneity of the patient cohort, potentially affecting postoperative outcomes. Standardizing surgical approaches in future studies will be essential to minimize this variability and to improve the consistency of the result.

Third, for patients without obesity, it is neither feasible nor ethical to obtain omental tissue from metabolically healthy individuals who are not undergoing surgery. Therefore, we enrolled patients with stage I EGC who had not received any inflammation-inducing drugs or procedures. EGC is typically characterized by a slow and biologically indolent course, remaining asymptomatic for prolonged periods without notable weight loss or energy wasting attributable to tumor state, and is frequently detected incidentally during screening rather than through symptom presentation [35–38], while clinical manifestations such as weight loss or abdominal pain usually appear only in advanced stages [47], a pattern consistently reported across both Eastern and Western cohorts. Furthermore, although obesity has been implicated in carcinogenesis through activation of the insulin-phosphoinositide 3-kinase (PI3K) signaling axis [48], or maintenance of chronic low-grade inflammatory circumstance through immune cells and cytokines, including adipokines [49], evidence for the reverse relationship that cancer itself induces obesity or metabolic dysregulations remains scarce. Therefore, patients with stage I EGC without obesity were considered an ethically acceptable and metabolically stable control group for investigating adipose tissue biology.

Fourth, the subgroup analysis within the experimental group demonstrated limited statistical power due to the small number of patients in each subgroup. While the results are primarily descriptive and exploratory, they suggest potential trends that may help generate hypotheses for future studies with larger and more balanced cohorts.

Lastly, the assessment of macrophage polarization requires surgically excised visceral adipose tissue, which can be obtained exclusively during bariatric surgery, limiting its feasibility as a preoperative predictive parameter. Therefore, further research focusing on laboratory markers is necessary to identify alternative preoperative surrogate markers.

## Conclusions

This study is the first to analyze macrophage distribution in visceral adipose tissue obtained from the patients with obesity and diabetes undergoing bariatric surgery. The findings revealed significant macrophage accumulation with M1 macrophage dominance with proinflammatory microenvironment, indicating a potential role in insulin resistance and diabetes progression. However, notable heterogeneity was observed within the patients with obesity and diabetes, as patients with a relatively higher degree of M1 dominance with proinflammatory microenvironment tended to demonstrate better postoperative diabetic remission. This suggest that while the chronic inflammatory circumstance driven by M1 macrophages in visceral adipose tissue is the main mechanism of diabetes progression, a relatively active inflammatory response may potentially indicate the presence of metabolically salvageable tissue still undergoing injury. Such ongoing tissue inflammation may paradoxically reflect the potential reversibility of the remaining beta-cell component and may be associated with an increased likelihood of postoperative diabetic remission before irreversible damage occurs, whereas a “silent” or diminished inflammatory response may rather indicate an already irreversible fibrotic profile of beta-cell loss.

Overall, this study provides novel human evidence linking macrophage polarization in visceral adipose tissue to metabolic status and postoperative outcomes in obesity and diabetes. Further studies with larger cohorts and randomized controlled trials are needed to validate these findings and clarify the causal relationship between macrophage polarization and metabolic outcomes.

## Supplementary Information

Below is the link to the electronic supplementary material.

Supplementary Material 1 (PPTX 383 KB)

## Data Availability

The datasets generated and analysed during this study are not publicly available due to institutional restrictions and patient confidentiality regulations.
