# Supplementary material for: Association of Macrophage Accumulation and Polarization in Patients with Obesity and Diabetes with Diabetic Remission After Bariatric Surgery
Source: Obes Surg. 2025 Dec 22;36(2):404–17. doi: 10.1007/s11695-025-08389-0 (PMC12957124; doi:10.1007/s11695-025-08389-0)
Supplement: Supplementary file 1 — Supplementary Material 1 (PPTX 383 KB) [file 11695_2025_8389_MOESM1_ESM.pptx]

## Slide 1
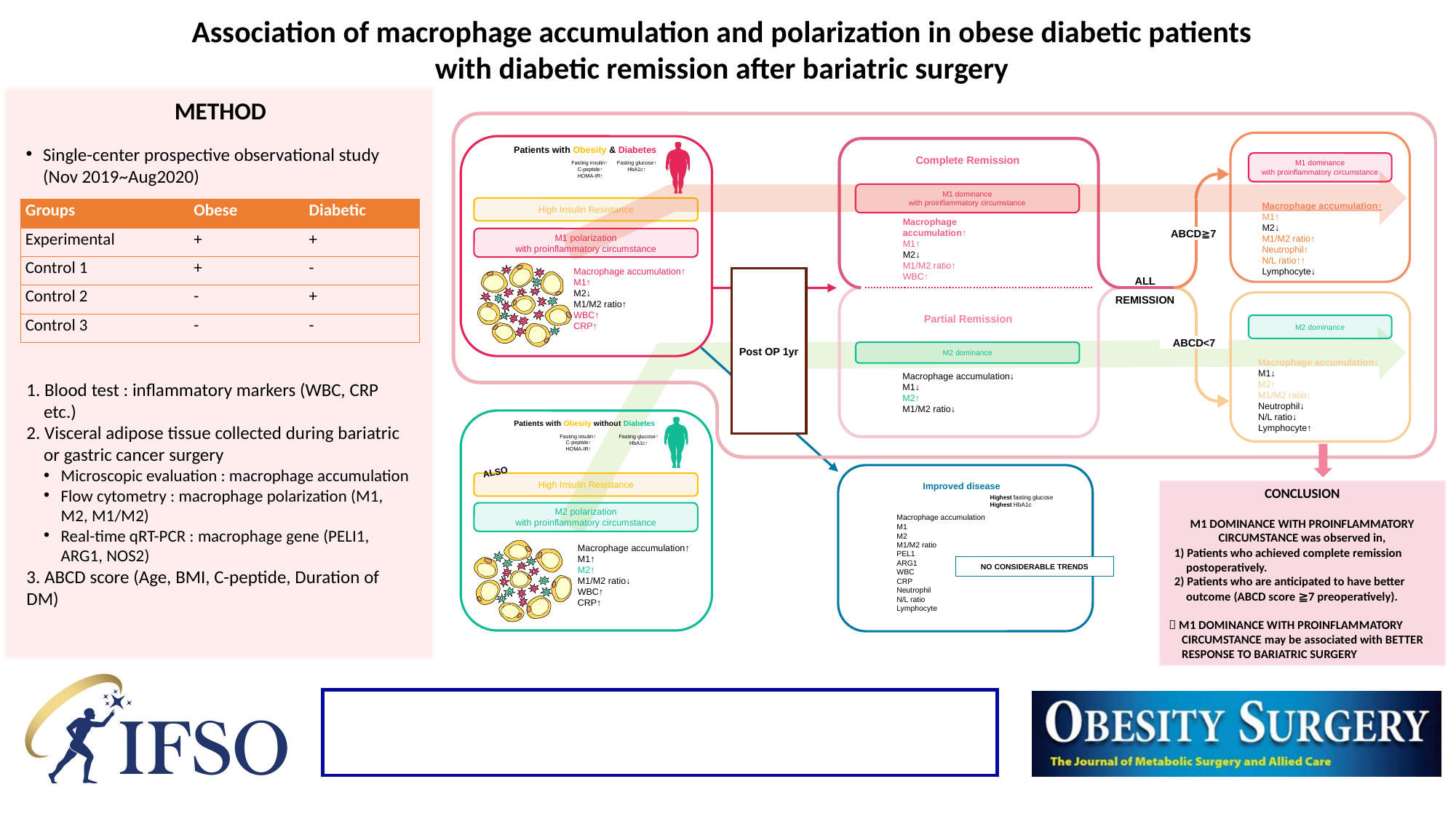

Association of macrophage accumulation and polarization in obese diabetic patients
with diabetic remission after bariatric surgery
METHOD
Patients with Obesity & Diabetes
Fasting insulin↑
C-peptide↑
HOMA-IR↑
Fasting glucose↑
HbA1c↑
Complete Remission
M1 dominance
with proinflammatory circumstance
M1 dominance
with proinflammatory circumstance
 Macrophage accumulation↑
 M1↑
 M2↓
 M1/M2 ratio↑
 Neutrophil↑
 N/L ratio↑↑
 Lymphocyte↓
High Insulin Resistance
Macrophage accumulation↑
M1↑
M2↓
M1/M2 ratio↑
WBC↑
ABCD≧7
M1 polarization
with proinflammatory circumstance
Macrophage accumulation↑
M1↑
M2↓
M1/M2 ratio↑
WBC↑
CRP↑
ALL
REMISSION
Post OP 1yr
Partial Remission
M2 dominance
ABCD<7
M2 dominance
 Macrophage accumulation↓
 M1↓
 M2↑
 M1/M2 ratio↓
 Neutrophil↓
 N/L ratio↓
 Lymphocyte↑
Macrophage accumulation↓
M1↓
M2↑
M1/M2 ratio↓
Patients with Obesity without Diabetes
Fasting insulin↑
C-peptide↑
HOMA-IR↑
Fasting glucose↑
HbA1c↑
ALSO
High Insulin Resistance
Improved disease
Highest fasting glucose
Highest HbA1c
M2 polarization
with proinflammatory circumstance
Macrophage accumulation
M1
M2
M1/M2 ratio
PEL1
ARG1
WBC
CRP
Neutrophil
N/L ratio
Lymphocyte
Macrophage accumulation↑
M1↑
M2↑
M1/M2 ratio↓
WBC↑
CRP↑
NO CONSIDERABLE TRENDS
Single-center prospective observational study (Nov 2019~Aug2020)
| Groups | Obese | Diabetic |
| --- | --- | --- |
| Experimental | + | + |
| Control 1 | + | - |
| Control 2 | - | + |
| Control 3 | - | - |
1. Blood test : inflammatory markers (WBC, CRP etc.)
2. Visceral adipose tissue collected during bariatric or gastric cancer surgery
Microscopic evaluation : macrophage accumulation
Flow cytometry : macrophage polarization (M1, M2, M1/M2)
Real-time qRT-PCR : macrophage gene (PELI1, ARG1, NOS2)
3. ABCD score (Age, BMI, C-peptide, Duration of DM)
CONCLUSION
M1 DOMINANCE WITH PROINFLAMMATORY CIRCUMSTANCE was observed in,
 1) Patients who achieved complete remission postoperatively.
 2) Patients who are anticipated to have better outcome (ABCD score ≧7 preoperatively).
 M1 DOMINANCE WITH PROINFLAMMATORY CIRCUMSTANCE may be associated with BETTER RESPONSE TO BARIATRIC SURGERY
